# Pharmacological semen collection in domestic and wild canids and felids: literature review

**DOI:** 10.1590/1984-3143-AR2023-0036

**Published:** 2024-02-02

**Authors:** Jeandson da Silva Carneiro, Tathiana Ferguson Motheo

**Affiliations:** 1 Universidade de Cuiabá, Cuiabá, MT, Brasil

**Keywords:** α_2_ adrenergic agonists, electroejaculation, reproduction, conservation

## Abstract

Semen collection methods vary greatly and rely on the practitioner's expertise, available materials, and the specific behavioral traits of the male animals involved. When it comes to domestic cats, wild felids, and canids, semen collection is particularly challenging. Thus, given the difficulty of semen collection in these species, pharmacological semen collection (PSC) stands out since it is a quick and straightforward method that does not require specific equipment. The PSC consists of administering α2-adrenergic receptor agonist drugs, mainly medetomidine, and dexmedetomidine, aiming semen release into the urethra with posterior urethral catheterization and sperm recovery. This technique was primarily described in domestic cats and wild felids, and despite the decreased seminal volume, the retrieved semen is highly concentrated and presents good quality. However, further studies are required to optimize semen collection in domestic dogs and wild canids. Therefore, the purpose of this review is to provide a comprehensive overview of the research developed on pharmacological semen collection (PSC) in the past few decades. The objective is to equip professionals with the essential knowledge required for the efficient application of this technique in both domestic and wild canids and felids and to make a valuable contribution to conservation efforts and the preservation of biodiversity, aligning with the principles of One Conservation.

## Introduction

In recent decades, there has been a notable surge in the interest in feline and canine seminal collection and evaluation. Domestic dogs and cats hold immense significance as experimental models for advancing and refining reproductive biotechnologies, mainly targeted toward their wild counterparts (Franklin et al., 2018; Jorge-Neto et al., 2020).

Semen collection methods are diverse and highly dependent on the practitioner's skills, the available materials, and the behavioral characteristics of the males depending on the species. In dogs, digital manipulation is the method of choice and provides good-quality semen. However, it requires conditioning before collection (Kutzler, 2005).

Electroejaculation (EE) is routinely performed in domestic and wild felids and canids. Nevertheless, apart from the essential requirements of specialized EE equipment and a skilled practitioner, this technique demands higher doses of anesthesia. This measure serves the dual purpose of mitigating any potential discomfort caused by the equipment and ensuring the safety of the team (Araujo et al., 2018). As a result, EE provides a quite diluted semen sample with a considerable amount of seminal plasma and a pronounced susceptibility to urine contamination. In many instances, centrifugation is imperative to rectify the concentration (Zambelli and Cunto, 2006; Zambelli et al., 2008; Araujo et al., 2018, 2020, 2021).

As an alternative to EE, pharmacological semen collection (PSC) was initially described by Zambelli et al. (2008). It is currently employed for semen retrieval in both domestic and wild felids and canids. This technique offers several advantages over EE, including the requirement for less profound anesthesia, rapid sedative recovery, and the possibility of reversal. It also provides a smaller semen volume, however, with a higher sperm concentration and absence of urine contamination. Notably, the decreased volume of seminal plasma eliminates the need for sample centrifugation, rendering it particularly advantageous when dealing with free-living animals (Zambelli et al., 2008; Kheirkhah et al., 2017; Swanson et al., 2017; Franklin et al., 2018; Kuczmarski et al., 2020).

The underlying principle of PSC involves administering selective α2-adrenergic agonist drugs, which lead to the contraction of the smooth muscle of the *ductus deferens* and consequent semen release through the urethra. Semen is retrieved by capillarity using a tomcat-type semi-rigid urethral catheter with an open end. This approach eliminates the need for catheters with side openings and ensures efficient and reliable semen collection (Zambelli et al., 2008; Silva et al., 2022).

Among the α2-adrenergic agonist drugs, there are reports of the use of detomidine, xylazine, medetomidine, and dexmedetomidine, with the latter two yielding the best results (Zambelli et al., 2008; Kheirkhah et al., 2017; Swanson et al., 2017; Franklin et al., 2018; Kuczmarski et al., 2020; Silva et al., 2022). Medetomidine is a drug with a high α2/α1 selectivity ratio of 1620/1, making it 6.2 times more selective for α2-adrenergic receptors than detomidine and ten times more selective than xylazine (Virtanen et al., 1988). Dexmedetomidine, on the other hand, is the dextrorotatory enantiomer of medetomidine, which has the same selectivity ratio for α2-adrenergic receptors and low affinity for β-adrenergic, muscarinic, dopaminergic, serotonergic, opioid, and γ-aminobutyric acid (GABA) receptors. As a result, it requires a lower dosage, thereby reducing undesirable side effects (Gerresheim and Schwemmer, 2013; Julião and Abimussi, 2019).

During semen and oocytes collection from *Panthera onca* and *Puma concolor,*Luczinski et al. (2020) observed the occurrence of heart murmurs, mitral valve regurgitation, and mild tricuspid regurgitation when employing medetomidine (100 μg/kg; im) and ketamine (5 mg/kg; im) for chemical restraint. Similarly, Carvalho et al. (2019) reported minor valve insufficiencies in domestic cats subjected to dexmedetomidine treatment (5 µg/kg; im).

Furthermore, Romagnoli et al. (2016) observed reduced heart rate, increased cardiac preload, and compromised systolic function in domestic cats after medetomidine administration (130 μg/kg; im). Diggelmann et al. (2023) assessed cardiological changes in this species, focusing on cardiac troponin I (cTnI) levels, following medetomidine treatment (100 μg/kg; im). However, they found that atipamezole (5 μg/kg; im) effectively restored hemodynamic parameters without a significant increase in cTnI concentrations during sedation reversal. Therefore, when monitored in healthy animals under veterinary supervision and followed by atipamezole reversal these transient cardiological changes do not pose significant risks to the animals.

In domestic cats, intramuscular administration of medetomidine at a dose of 130 µg/kg has a 3724.2 mL/kg volume distribution volume and an 11.9 mL/kg/minute clearance rate. Furthermore, it takes an average of 40 minutes to reach a serum concentration peak of 32.8 ng/mL. However, between 20 and 90 minutes after administration, the serum concentration of medetomidine remains stable at approximately 28 ng/mL. This indicates that during this period, additional semen collections are possible in case of unsuccessful attempts during the initial stages (Romagnoli et al., 2020).

Furthermore, anesthesia with dexmedetomidine or medetomidine allows its reversal with yohimbine or atipamezole (α2-adrenergic antagonists), making them safe and easily controllable anesthetics. These reversal agents enable the animals to be released approximately 30 minutes after their administration; however, they should be administered at least 40 minutes after ketamine application. This ensures staff safety and reduces the risk of complications associated with deepening anesthesia when using other drugs. Additionally, when working with free-living wild animals, releasing them soon after the procedures helps avoid potential issues arising from changes in weather conditions, such as sudden temperature drops and rainfall (Romagnoli et al., 2020; Araújo et al., 2021; Silva et al., 2022).

Hence, the main goal of this review is to synthesize the extensive research conducted over the past decades on PSC in an effort to improve the success of professionals in the use of this technique in both domestic and wild canids and felids. By leveraging the knowledge of the most effective PSC protocols for wild animals, this study also seeks to make significant contributions to conservation and biodiversity, embracing the concept of One Conservation (Pizzutto et al.*,* 2021a, b).

## Pharmacological semen collection in domestic cats

Zambelli et al. (2008) were the first to describe the pharmacological semen collection in domestic cats through a comparative study evaluating the quality and fertilization capacity of frozen-thawed semen collected by electroejaculation (EE) and pharmacological semen collection after the administration of medetomidine (130-140 μg/kg, im). When comparing the two collection methods, pharmacological induction followed by urethral catheterization using a tomcat-type 3 FR catheter (guided through the urethra approximately 9 cm) allowed the retrieval of good-quality semen samples. These samples were characterized by a smaller volume, higher sperm concentration, and lower pH compared to EE (Table 1). Regarding the cleavage rate, as well as the embryo production from fertilized oocytes using frozen-thawed semen, there were no significant differences between the two semen collection methods employed.

**Table 1:** Research on pharmacological semen collection (PSC) in domestic cats.

**Reference**	**Species**	Method^*^	**Dosage/drug**	Vol^*^ (μL)	Conc^*^ (sperm/mL)	Total sperm (n)^*^	Mot (%)^*^	Prog Mot^*^
Zambelli et al. (2008)	Domestic cat (*Felis catus)*	PSC	130-140 μg/kg Medetomidine	10.5	1868.4 (10^6^)	21 (10^6^)	78.1	4.7
		EE	-	67.1	542.9 (10^6^)	33.6 (10^6^)	78.1	4.5
Zambelli et al. (2010)	Domestic cat (*Felis catus)*	PSC	130-140 μg/kg Medetomidine	20.9	1453.3 (10^6^)	30.3 (10^6^)	59.5	3.5
		EE	-	89	223.8 (10^6^)	19.9 (10^6^)	66.9	4.1
Filliers et al. (2010)	Domestic cat (*Felis catus)*	PSC	100 μg/kg Medetomidine + 5 mg/kg Ketamine	-	-	-	50.4	-
		ES	-	-	-	-	71.5	-
Prochowska et al. (2015)	Domestic cat (*Felis catus)*	PSC	100 μg/kg Medetomidine	15.5	3257.8 (10^6^)	47.7 (10^6^)	64	-
		ES	-	-	-	52.9 (10^6^)	46.6	-
Cunto et al. (2015)	Domestic cat (*Felis catus)*	PSC	130 μg/kg Medetomidine	10.6	1753 (10^6^)	19.9 (10^6^)	63	4
		PSC	50 μg/kg Medetomidine	5.6	215 (10^6^)	0.9 (10^6^)	49	3
Prochowska et al. (2016)	Domestic cat (*Felis catus)*	PSC	100 μg/kg Medetomidine	-	-	57.25 (10^6^)	75	-
		ES	-	-	-	53.67 (10^6^)	62.5	-
Swanson et al. (2017)	Domestic cat (*Felis catus)*	PSC	1 mg/kg Xylazine + 10 mg/kg Ketamine	13	-	0.1 (10^6^)	-	-
		PSC	25 μg/kg Dexmedetomidine + 10 mg/kg Ketamine	25	-	33.6 (10^6^)	-	-
		EE	1 mg/kg Xylazine + 10 mg/kg Ketamine	104	-	27.3 (10^6^)	-	-
		EE	25 μg/kg Dexmedetomidine + 10 mg/kg Ketamine	78	-	19.9 (10^6^)	-	-
Madrigal-Valverde et al. (2021)	Domestic cat (*Felis catus*	PSC	100 µg/kg Medetomidine + 5 mg/kg Ketamine	40	667.55	~4 (10^6^)	81.67	3.75-
		PSC	25µg/kg Dexmedetomidine + 5 mg/kg Ketamine	33.75	181.75	~4 (10^6^)	81.25	3.75
Silva et al. (2021)	Domestic cat (*Felis catus)*	PSC	100 μg/kg Medetomidine + 5 mg/kg Ketamine	10.56	3.24 (10^9^)	21.69 (10^9^)	71.7	4.11
		PSC	250 μg/kg Detomidine + 5 mg/kg Ketamine	8.88	2.15 (10^9^)	12.77 (10^9^)	49.8	3.1
Hidalgo et al. (2023)	Domestic cat (*Felis catus)*	PSC 10’	25µg/kg Dexmedetomidine + 5 mg/kg Ketamine	22.62	48.10 (10^6^)		67	2.4
		PSC 15’		26.81	90.18 (10^6^)		81.87	3.0

* Vol: Volume; Conc: Concentration; sperm: Spermatozoa; Mot: Total motility; Prog Mot: Progressive motility; PSC: Pharmacological semen collection; ES: Epidydimal slicing; EE: Eletroejaculation.

Furthermore, the same authors aimed to compare cat seminal plasma composition, protein profile (with SDS-page), and protein and zinc concentrations from ejaculates obtained by PSC and EE. They described that the recovery method influences the seminal plasma protein profile once EE was related to the absence of two proteins (P55 and P14) and the alteration of three protein bands (P200, P80, P28). Therefore, these findings suggested a potential correlation with microtraumas induced in the urethra during catheterization. Additionally, the collection method also affected zinc and protein concentrations, and these parameters were significantly higher in samples collected by PSC (Zambelli et al., 2010).

In a study conducted by Cunto et al. (2015), the effects of two different doses of medetomidine on semen parameters were examined. The study compared a 130 μg/kg dose with a 50 μg/kg dose of medetomidine administered intramuscularly (im) per PSC, using a 3 FR urinary catheter. The results revealed that the group receiving 130 μg/kg of medetomidine exhibited improved semen characteristics, including higher semen volume, concentration, total sperm count, total and progressive motilities when compared to the 50 μg/kg group (Table 1). Moreover, it was observed that 50 μg/kg of medetomidine failed to provide sufficient sedation, as indicated by involuntary movements in the hind limbs during the procedure. Thus, the authors suggested that by administering 130 μg/kg of medetomidine, high-quality semen can be reliably obtained through a single collection immediately after the drug's effects have subsided.

Hidalgo et al. (2023) assessed the effects of dexmedetomidine (30 μg/kg, im) combined with ketamine (5 mg/kg, im) on domestic cats for pharmacological semen collection. The study examined two collection time points, 10 minutes and 15 minutes after anesthesia. The results revealed that semen collected at the 15-minute mark exhibited a greater volume, higher concentration, and lower percentage of minor defects than the 10-minute collection. Regarding sperm kinetics, the 15-minute mark showed better total motility and faster cells (FAST), whereas a higher percentage of sperm cells with slow speed (SLOW) were seen at the 10-minute mark. Thus, this suggests that the optimal outcome for pharmacological collection using dexmedetomidine is achieved when catheterization is performed 15 minutes after drug administration.

In a study conducted by Madrigal-Valverde et al. (2021), the effects of medetomidine (100 µg/kg, im) and dexmedetomidine (25 µg/kg, im), both in association with ketamine (5 mg/kg), were compared using a 1 mm in diameter and 13 cm in length urethral catheter in domestic cats undergoing pharmacological semen collection. The authors aimed to evaluate seminal parameters, including volume, concentration, sperm vigor, percentage of total and progressive motility, percentage of morphologically normal spermatozoa, and the structural and functional integrity of the plasmatic membrane (Table 1). Surprisingly, the results indicated no significant differences in seminal parameters between the two groups, suggesting that both medetomidine and dexmedetomidine, when combined with ketamine, have comparable effects on seminal parameters in domestic cats undergoing PSC. Nevertheless, the authors concluded that dexmedetomidine offers greater advantages over medetomidine due to its cost-effectiveness and serves as a viable alternative in countries where the sale of medetomidine is prohibited.

Silva et al. (2021) compared the results of PSC using detomidine (250 μg/kg; im) *versus* medetomidine (100 μg/kg, im) associated with ketamine (5 mg/kg, im). A tomcat-type (3F) urethral catheter was placed (7 – 9 cm) through the urethra 20 minutes after induction, and both protocols retrieved semen. The semen collected after the medetomidine administration exhibited higher concentration and better seminal quality than semen obtained with detomidine (Table 1). Additionally, there was considerable urethral resistance after detomidine treatment, which was attributed to the lower affinity of detomidine for α2-adrenergic receptors that probably resulted in insufficient activation of these receptors, leading to inadequate relaxation of the *ductus deferens* and poor semen release into the urethra.

As an alternative to the induction with dexmedetomidine (25 μg/kg; im), Swanson et al. (2017) evaluated the potential use of xylazine (1 mg/kg; im). A 3.5 FR urethral catheter (9 cm deep into the urethra) was used for semen retrieval. However, despite also being an α2-agonist, xylazine did not yield satisfactory results, which could be attributed to its 10-fold lower specificity for α2-adrenergic receptors compared to dexmedetomidine (Table 1).

Prochowska et al. (2015) collected samples from 214 tomcats to compare basic semen characteristics and sperm motility parameters via computer-assisted sperm analysis (CASA) after pharmacological semen collection *versus* epididymal slicing (ES). For PSC, medetomidine (100 μg/kg; im) was administered, and semen was collected twice after 10 minutes with a tomcat-type catheter (3F, 9cm into the urethra). Shortly after, ketamine (5 mg/kg; im) and meloxicam (0.3 mg/kg; sc) were administrated, and an orchiectomy was performed. Epididymal sperm cells were recovered by ES 5 - 10 minutes after the testis removal. Thus, semen collected by PSC and by ES had similar characteristics according to total sperm count, subjective motility, membrane integrity, and morphology (Table 1). However, the authors suggested that despite the comparable characteristics of ES and PSC semen, the latter appears to be better suited for analysis using CASA than ES samples.

Regarding the effectiveness of *in vitro* fertilization using semen retrieved by the PSC protocol compared to ES, Zambelli et al. (2008) described that both semen collection methods provided high-quality samples suitable for this purpose. Furthermore, these methods exhibited comparable characteristics following the freezing/thawing process, making them valuable techniques for banks of genetic resources of semen (Filliers et al., 2010; Prochowska et al., 2016; Prochowska and Niżański, 2017).

Given the aforementioned studies, it can be concluded that the most effective approach for pharmacological semen collection in domestic cats involves the use of a tomcat-type 3 FR urethral catheter inserted at a 9 cm depth into the urethra featuring an open end without side openings. The procedure includes two to three catheterizations promptly following the administration of 100 to 140 μg/kg of medetomidine or 25 μg/kg dexmedetomidine, intramuscularly.

## Pharmacological semen collection in wild felids

Assisted reproductive techniques play a vital role in species conservation. These techniques aim to enhance genetic diversity among individuals, both in their natural habitats (*in situ*) and controlled environments (*ex situ*). Semen collection represents a critical step in these methods, requiring samples of sufficient quantity and quality for successful implementation (Jorge-Neto et al., 2019; Araujo et al., 2020). Considering the well-succeeded application of the PSC protocol in domestic cats, its adaptation and refinement in wild felids have been pursued to achieve the most favorable outcomes.

Aiming to evaluate the effectiveness of PSC in African lions (*Panthera leo*), Lueders et al. (2012) administered medetomidine (12 mg; im) combined with ketamine (150 mg; im). Catheterization was performed using a 2.6 x 500 mm or 3.3 x 500 mm canine urethral catheter, 25 to 35 cm deep in the urethra, between 20 to 40 minutes after the pharmacological induction. The recovered semen showed high concentration, motility, and longevity (Table 2).

**Table 2:** Research on pharmacological semen collection (PSC) in wild felines.

**Reference**	**Species**	Method*	**Dosage/drug**	Vol^*^ (μL)	Conc^*^ (sperm/mL)	Total sperm (n)^*^	Mot (%)^*^	Prog Mot^*^
Lueders et al. (2012)	African lion (*Panthera leo)*	PSC	12 mg Medetomidine + 150 mg Ketamine	422	1.94 (10^9^)	1.14 (10^9^)	79.50	-
Lueders et al. (2013)	African lion (*Panthera leo)*	PSC	12 mg Medetomidine + 150 mg Ketamine	100- 1000	130-4860 (10^6^)	-	62.5- 97.2	35-96%
	Cheetah (*Ancinonyx jubatus*)	PSC	2 mg Medetomidine + 80 mg Ketamine	50-100	0-455 (10^6^)	-	0-60	0-45%
	Leopard (*Panthera pardus*)	PSC	2 mg Medetomidine + 10 mg Midazolam + 15 mg Butorphanol	250	635 (10^6^)	-	92	59%
	Snow leopard (*Panthera uncia*)	PSC	1.5 mg Medetomidine + 100 mg Ketamine	500	3.1 (10^6^)	-	40	10%
	Tiger (*Panthera tigris*)	PSC	4 mg Medetomidine + 120 mg Zoletil^®^	50-300	0-2500 (10^6^)	-	0-84.6	0-67%
	Asian golden cat (*Catopuma temminckii*)	PSC	0.8 mg Medetomidine + 75 mg Ketamine	250	70.6 (10^6^)	-	65	35%
Kheirkhah et al. (2017)	Jungle cat (*Felis chaus*)	PSC	1 mg Medetomidine + 5mg Ketamine	69	75.13 (10^6^)	-	-	-
Jeong et al. (2018)	Amur leopard *(P. bengalensis euptilurus)*	PSC	50 μg/kg Medetomidine + 4 mg/kg Ketamine	6.7	1698 (10^6^)	-	84.1	-
Araujo et al. (2018)	Jaguar (*Panthera onca)*	PSC	80-100 μg/kg Medetomidine + 5 mg/kg Ketamine	347	2635 (10^6^)	745 (10^6^)	77	3.75
Jorge-Neto et al. (2019)	Jaguar (*Panthera onca*)	PSC	80-100 μg/kg Medetomidine + 5 mg/kg Ketamine	347.2	2635.2 (10^6^)	-	77	3.75
	Cougars (*Puma concolor)*	PSC	80-100 μg/kg Medetomidine + 5 mg/kg Ketamine	205	749.4 (10^6^)	-	72.5	3.25
	Ocelot (*Leopardus pardalis*)	PSC	80-100 μg/kg Medetomidine + 5 mg/kg Ketamine	27	1670 (10^6^)	-	90	5
	Jaguarundi (*Herpailurus yagouaroundi*)	PSC	80-100 μg/kg Medetomidine + 5 mg/kg Ketamine	20	570 (10^6^)	-	60	3
	Margay (*L. wiedii*)	PSC	80-100 μg/kg Medetomidine + 5 mg/kg Ketamine	20	120 (10^6^)	-	90	4
	Southern tiger cat (*L. guttulus*)	PSC	80-100 μg/kg Medetomidine + 5 mg/kg Ketamine	23	1720 (10^6^)	-	80	4
Araujo et al. (2020)	Cougar (*Puma concolor)*	PSC	80-100 μg/kg Medetomidine + 5 mg/kg Ketamine	106.7	524 (10^6^)	56.5 (10^6^)	70	3
		EE	1.2 mg/kg Xylazine + 10 mg/kg Ketamine	450	205 (10^6^)	93.5 (10^6^)	75	3.5
Jorge-Neto et al. (2020)	Jaguar (*Panthera onca)*	PSC	80-100 μg/kg Medetomidine + 5 mg/kg	297.5	359 (10^6^)	139.5 (10^6^)	3	1
Iglesias et al. (2020)	Southern tiger cat (*Leopardus guttulus)*	PSC	8 μg/kg Dexmedetomidine + 10 mg/kg Ketamine	35.9	552 (10^6^)	-	71	3.1
Jorge-Neto et al. (2023)	Jaguar (*Panthera onca*)	PSC	80-100 μg/kg Medetomidine + 5 mg/kg Ketamine	117.8	2344 (10^6^)	207 (10^6^)	55.3	36.2%

* Vol: Volume; Conc: Concentration; sperm: Spermatozoa; Mot: Total motility; Prog Mot: Progressive motility; PSC: Pharmacological semen collection; ES: Epidydimal slicing; EE: Eletroejaculation.

Additionally, the authors emphasized the absence of urine and reduced presence of seminal plasma in the samples, which are crucial attributes of the PSC method, and ensure the suitability of semen for assisted reproductive techniques. To prevent urine contamination, it was advised not to insert the catheter more than 30 cm into the urethra of an adult lion. Furthermore, they highlighted a positive correlation between increased semen quantity and the application of rectal massage and prostate stimulation during transrectal ultrasound procedures.

In a study conducted by Lueders et al. (2013), the authors assessed the variation in pharmacological semen collection response in six different wild cats, namely lion (*Panthera leo)*, cheetah (*Acinonyx jubatus*), African leopard (*Panthera pardus*), snow leopard (*Panthera uncia*), tiger (*Panthera tigris*), and Asian golden cat (*Catopuma temminckii*). To induce PSC, the authors administered medetomidine at varying doses (12, 2, 2, 1.5, 4 and 0.8 mg/animal, respectively) in association with other anesthetics like ketamine, butorphanol, midazolam, and zolazepam combined with tiletamine (Zoletil^®^). Catheterization was performed using urethral catheters with diameters ranging from 1.3 to 3.3 mm and depths between 12 and 40 cm, depending on the species under investigation. The results obtained from this collection technique yielded successful outcomes, with small semen volumes ranging from 50 to 1000 µL and high sperm concentrations, which varied according to the species (Table 2). These findings were attributed to the decreased seminal plasma content compared to EE ejaculates. Therefore, these samples could be directly employed for intrauterine artificial insemination and cryopreservation.

Another study verified the pharmacological semen collection viability in Jungle cats (*Felis chaus*) after induction with medetomidine (1 mg; im) and ketamine (5 mg; im). Mature males were isolated from females for at least of three days prior to the procedure to prevent mating, ensuring an empty *ductus deferens*. Semen was collected using a 2 mm tomcat-type urethral catheter inserted 12 to 14 cm into the urethra approximately 30 minutes after drug administration. Retrieved semen presented low contamination a high concentration, motility, and viability (Table 2).

Thus, based on the quality of semen collected through PSC in *Felis chaus*, the authors suggested the use of this technique for long-term semen preservation purposes, such as genetic resource banks (Kheirkhah et al., 2017).

Jeong et al. (2018) conducted a study to assess the effectiveness of the PSC method in six Amur leopard cats (*Prionailurus bengalensis euptilurus*). The authors administered medetomidine (50 μg/kg; im) with ketamine (4 mg/kg; im) to induce sedation. After 20 minutes, catheterization was performed using a 1 mm tomcat-type urethral catheter. The retrieved semen exhibited a small volume and high concentration (Table 2) and suitable quality for artificial insemination in these animals. Additionally, the authors highlighted several advantages associated with the use of PSC, including reduced time, cost, invasiveness, and less likelihood of urine contamination compared to electroejaculation (EE).

In jaguars (*Panthera onca*), Araujo et al. (2018) aimed to compare the seminal parameters of captive and free-living animals used medetomidine (80–100 μg /kg; im) in association with ketamine (5 mg/kg; im) for sedation. Urethral catheterization was performed with a tomcat-type probe inserted 1 mm to 12 cm deep into the urethra. Retrieved semen samples presented good quality, with adequate sperm concentration for application in assisted reproductive techniques (Table 2). However, this parameter was found to be lower in captive jaguars when compared to their free-living counterparts, and the authors attributed this difference to nutritional factors that impact animals kept in captivity.

Jorge-Neto et al. (2020) reported a successful semen collection from a free-living jaguar with cryptorchidism after chemical restraint using medetomidine (0.01 mg/kg; im) and ketamine (5 mg/kg; im). The male jaguar was captured on two separate occasions, and in both instances, semen was retrieved using a tomcat-type urethral catheter (with an open end, 3 FR, 130 mm long), and sperm parameters were subjectively assessed. Thus, in both captures, semen presented poor quality, with low concentration and decreased total sperm count for the species (Table 2).

In a study conducted by Jorge-Neto et al. (2019), the authors submitted six Brazilian wild cats, including *Panthera onca*, *Puma concolor*, *Leopardus pardalis*, *Herpailurus yagouaroundi*, *Leopardus wiedii*, *Leopardus guttulus*, to pharmacological semen collection. The anesthetic protocol employed involved the administration of medetomidine (80-100 μg/kg; im) associated with ketamine (5 mg/kg; im). After a 15 to 20-minute interval, catheterization was performed using a tomcat-type urethral probe with an open end (without side openings), 14 cm long for medium and large cats and 7 - 9 cm long for small cats. Each animal underwent two catheterizations: the first involving catheter insertion alone and the second incorporating transrectal prostate massage (Table 2). Transrectal prostate massage was crucial for increasing seminal volume and sperm concentration in Brazilian wildcats without impairing seminal quality (vigor and motility) or its subsequent cryopreservation (Jorge-Neto et al., 2019). Despite exhibiting a lower seminal volume than other collection methods employed in felines, the concentration and total number of spermatozoa were notably higher. This observation suggested the presence of minimal amounts of seminal plasma in the samples, which enables PSC in free-living animals once it yields good-quality semen, simplifies sample manipulation in the field, and eliminates the need for semen centrifugation (Jorge-Neto et al., 2019).

Araújo et al. (2020) conducted a comparative study between two semen collection techniques, namely pharmacological semen collection (PSC) and electroejaculation (EE), in pumas (*Puma concolor*). The sedation protocol involved administering medetomidine (80-100 μg/kg; im) in combination with ketamine (5 mg/kg; im). Approximately 20 to 40 minutes after the administration, a tomcat-type urethral catheter was carefully inserted into the urethra, reaching a depth of 12 cm, for semen collection.

The results of the study revealed that the PSC technique produced seminal samples of similar quality to those retrieved through EE. However, it was noticed that PSC resulted in a lower semen volume and higher sperm concentration when compared to EE, (Table 2). The authors attributed these differences to the absence of urine contamination and the minimal presence of seminal plasma in PSC samples. Thus, this eliminates the need for centrifugation of the semen samples when using PSC. Additionally, the researchers highlighted the advantage of using atipamezole (0.25 mg/kg; im) for reversal, as it facilitates the recovery of animals *in situ*.

To determine the optimal dose of dexmedetomidine for PSC in *Leopardus guttulus*, Iglesias et al. (2020) evaluated different doses of dexmedetomidine (ranging from 3 μg/kg to 8 μg/kg; im) and ketamine (ranging from 8 mg/kg to 13.9 mg/kg; im) with immediate catheterization of the urethra, with a 3.5 FR tomcat type urethral catheter (7.5 cm into the urethra). The results indicated that 8 μg/kg of dexmedetomidine associated with 10 mg/kg of ketamine promoted the most favorable outcomes in sperm concentration, motility, vigor, sperm viability, and acrosome integrity (Table 2). Therefore, these findings firmly establish PSC as a well-suited and effective technique for semen collection in this species.

Prochowska et al. (2022) surveyed data on five years of semen collections performed on different species of wild cats (*Acinonyx jubatus, Leptailurus serval, Caracal caracal, Leopardus pardalis, Felis margarita, Lynx lynx, Panthera onca, Panthera pardus, Panthera leo, Otocolobus manul, Panthera uncia, Panthera tigris*), kept in zoos or on private properties in Poland, Ukraine, and Belgium. Various collection methods were employed, encompassing epidydimal slicing (ES), electroejaculation (EE), and pharmacological semen collection (PSC), primarily for artificial insemination attempts and infertility evaluation in these animals. PSC involved the administration of 100 – 150 µg/kg medetomidine, followed by urethral catheterization. In small felids such as sand cats, servals, and ocelots, a tomcat urinary catheter measuring 1.0 mm in diameter and 130 mm in length was employed for semen collection. However, a dog urinary catheter with dimensions of either 2.0 mm × 300 mm or 2.6 mm × 500 mm was used for larger felids. Out of the 22 collection attempts, successful semen retrieval was achieved in 15 cases, yielding a minimum of 1 × 10^6^ spermatozoa. Out of the five ES performed, only two presented successful semen recovery. The PSC technique demonstrated the ability to collect semen ranging from 30 to 236 × 10^6^ total sperm across the five cases. However, in four collections, the semen volume obtained through PSC ranged between 0 and 8.5 × 10^6^ total spermatozoa. Interestingly, one case showed a comparable number of spermatozoa between PSC and EE methods. In two cases where PSC failed to collect semen successfully, EE was necessary, resulting in a high total sperm count. Unfortunately, in the remaining cases, EE did not provide successful semen collection.

To assess the efficacy of three cryoprotectants, namely dimethyl sulfoxide (DMSO), glycerol (GLY), and methanol (MET) in the semen cryopreservation of jaguars (*Panthera onca*), Jorge-Neto et al. (2023) conducted a study using an association of ketamine (5 mg/kg; im) and medetomidine (0.01 mg/kg; im) for semen recovery. After urethral catheterization, 117.8 µL of semen was retrieved. Semen parameters were assessed via CASA, and fresh semen presented 55.3% total motility, 36.3% progressive motility, and 2344x10^6^ sperm/mL sperm concentration (Table 2). Post-thawed semen presented total motility rates of 5.28%, 4.49%, and 0.51% for DMSO, GLY, and MET, respectively. While no statistical significance was noticed between the DMSO and GLY groups, both outperformed MET in semen quality.

In conclusion, based on the aforementioned studies, the main protocols that provided higher-quality semen samples in wild cats involved the administration of medetomidine or dexmedetomidine associated with ketamine, followed by subsequent urethral catheterization after 20 to 40 minutes of induction.

## Pharmacological semen collection in domestic dog and wild canids

Kuczmarski et al. (2020) were the first to describe the PSC method in domestic dogs, using increasing doses of dexmedetomidine (5, 10, and 15 μg/kg; im) associated with ketamine (3 mg/kg, im). Urethral catheterization was performed 20 minutes after the administration, placing a urethral catheter (nº 6) 13 cm into the urethra of dogs weighing 5 to 10 kg. The study determined the dose of 15 μg/kg of dexmedetomidine associated with ketamine (3 mg/kg; im) as the minimum effective dose for semen retrieval in dogs (Table 3). However, urine contamination was observed in 44% of the samples, likely resulting from a catheter being deeply inserted. Therefore, the authors advised emptying the urinary bladder before seminal collection.

**Table 3:** Research on pharmacological semen collection (PSC) in wild canids.

**Reference**	**Species**	Method*	**Dosage/drug**	Vol^*^ (μL)	Conc^*^ (sperm/mL)	Total sperm (n)^*^	Mot (%)^*^	**Prog Mot**
Kuczmarski et al. (2020)	Domestic dog (*Canis familiaris*)	PSC	5 μg/kg Dexmedetomidine +3 mg/kg Ketamine	0	0	-	-	
		PSC	10 μg/kg Dexmedetomidine +3 mg/kg Ketamine	0	0	-	-	
		PSC	15 μg/kg Dexmedetomidine + 3 mg/kg Ketamine	90	1186.66 (10^6^)	114.9 (10^6^)	58.3	1.89
Lueders et al. (2013)	African wild dog (*Lycaon pictus*)	PSC	1 mg Medetomidine + 30 mg Zoletil	400	540 (10^6^)		93	82%
	Maned wolf (*Chrysocyon brachyurus*)	PSC	0.5 mg Medetomidine + 50 mg Ketamine + 5 mg Butorphanol	100	10 (10^6^)		40	30%
Franklin et al. (2018)	Red Wolf (*Canis rufus)*	PSC	40 μg/kg Medetomidine/ 20 μg/kg Dexmedetomidine + 0.4 mg/kg Butorphanol	0.36	50.4 (10^6^)	24.6 (10^6^)	38	~2.3
		EE	40 μg /kg Medetomidine/ 20 μg /kg Dexmedetomidine + 0.4 mg/kg Butorphanol	5.48	190 (10^6^)	720.3 (10^6^)	72	4.1
Silva et al. (2022)	Crab-eating fox (*Cerdocyon thous*)	PSC	100 μg/kg Medetomidine + 5 mg/kg Ketamine	39.13	277.5 (10^6^)	6.2 (10^6^)	40	2.57

* Vol: Volume; Conc: Concentration; sperm: Spermatozoa; Mot: Total motility; Prog Mot: Progressive motility; PSC: Pharmacological semen collection; EE: Eletroejaculation.

Lueders et al. (2013) assessed the efficacy of PSC in wild cats and two wild canids, namely *Lycaon pictus* and *Chrysocyon brachyurus*. The researchers administered medetomidine at 1 mg/animal and 0.5 mg/animal, respectively, in conjunction with other anesthetics, such as ketamine, butorphanol, and tiletamine associated with zolazepam (Zoletil^®^). For catheterization, a 2.6 mm diameter urethral catheter was inserted 30 - 35 cm deep into the urethra. Semen volume and concentration obtained from *Lycaon pictus* and *Chrysocyon brachyurus* were 400 µL, 540 x 10^6^ sperm/mL, and 100 µL, 10 x 10^6^ sperm/mL, respectively (Table 3). Similar to the findings described for the wild cats, the combination of a high concentration and small seminal volume indicates minimal seminal plasma, which makes this technique advantageous for direct applications such as intrauterine artificial insemination or cryopreservation.

In crab-eating foxes (*Cerdocyon thous*), successful semen collection was achieved in six adult males after treatment with medetomidine (100 μg/kg; im) associated with ketamine (5 mg/kg; im). Semen collection took place approximately 15 to 20 minutes after drug administration with a 4 FR tomcat-type catheter. The procedure was repeated every 10 minutes to retrieve additional samples until no semen was observed in the catheter. Comparisons were made between collections conducted during the reproductive and non-reproductive seasons. Significant variations in seminal parameters were observed between the two seasons, concluding that *Cerdocyon thous* males exhibits seasonal reproductive behaviors. Furthermore, the PSC protocol did not result in anesthetic complications and allowed the release of the animals after 30 minutes of yohimbine administration (0.4 mg/kg; im). Therefore, PSC proved to be an effective method for semen collection in this species (Table 3) (Silva et al., 2022).

To evaluate the efficacy of pharmacological semen collection in red wolves (*Canis rufus*), Franklin et al. (2018) used butorphanol (0.4 mg/kg; im) combined with either medetomidine (0.04 mg/kg; im) or dexmedetomidine (0.02 mg/kg; im). In certain cases, midazolam was administrated, and isoflurane was provided as necessary to maintain anesthesia. The outcomes obtained from this approach were compared with EE technique. Semen collection via urethral catheterization was performed between 20 to 30 minutes after induction, using a polypropylene catheter (3.5 FR x 22 in) inserted 26 cm into the urethra. Pharmacological semen collection exhibited a notable decline in sperm total and progressive motility, primarily due to urine contamination associated with this method. Furthermore, semen volume recovered through PSC was significantly smaller when compared to EE. Thus, based on these findings, the authors concluded that PSC is impractical for semen collection in this species (Table 3).

On the other hand, it appears that the dose of medetomidine (0.04 mg/kg) used in this study was lower than the 0.1 mg/kg of medetomidine used by Silva et al. (2022) in *Cerdocyon thous*. Furthermore, all animals underwent semen collection via PSC followed by EE, maintaining a consistent collection order without reversal. However, conducting two consecutive semen collections can potentially yield samples with compromised accuracy, as some semen may have been expelled during the initial collection. Therefore, employing the same collection sequence for all animals and using a lower dosage of the α2-adrenergic agonist may have prompted premature conclusions. Studies by González et al. (2023) with *Lynx canadensis* and Estrela et al. (2023) with *Puma yagouaroundi* also used sequential collection techniques.

To date, limited research has been conducted on the use of pharmacological semen collection (PSC) in both domestic and wild canids. While its viability has been established in domestic dogs and wild canids such as *Cerdocyon thous*, the efficacy of PSC in red wolves (*Canis rufus*) is not prominent. Therefore, additional studies are necessary to explore and evaluate the application and effectiveness of PSC in these species.

## Final considerations

Pharmacological semen collection has emerged as an effective and direct method for semen recovery, as it uses specific drugs and materials readily available in veterinary practice. This approach proves to be efficient and cost-effective, particularly as sedatives are commonly required for many procedures in wild animals.

By using α2-adrenergic agonists, this method serves the dual purpose of sedating and semen release into the urethra. In both domestic and wild dogs and cats, pharmacological semen collection yields good-quality semen samples with relatively low volume but high sperm concentration, which may be applied to various assisted reproductive techniques.

Finally, PSC requires less profound anesthesia and can be conveniently reversed with yohimbine or atipamezole, thereby reducing complications associated with sedation and minimizing animal discomfort compared to other methods. However, particularly in the case of wild canids, further studies are warranted to implement this semen collection technique.
